# Successful implementation of new technologies in nursing care: a questionnaire survey of nurse-users

**DOI:** 10.1186/1472-6947-11-67

**Published:** 2011-10-27

**Authors:** Anke JE de Veer, Margot AH Fleuren, Nienke Bekkema, Anneke L Francke

**Affiliations:** 1 The Netherlands Institute for Health Services Research (NIVEL), P.O. Box 1568, 3500 BN Utrecht, the Netherlands; 2 TNO, Leiden, the Netherlands; 3 Department of Public and Occupational Health, EMGO Institute for Health and Care Research (EMGO+) of the VU University Medical Center Amsterdam, the Netherlands

## Abstract

**Background:**

A growing number of new technologies are becoming available within nursing care that can improve the quality of care, reduce costs, or enhance working conditions. However, such effects can only be achieved if technologies are used as intended. The aim of this study is to gain a better understanding of determinants influencing the success of the introduction of new technologies as perceived by nursing staff.

**Methods:**

The study population is a nationally representative research sample of nursing staff (further referred to as the Nursing Staff Panel), of whom 685 (67%) completed a survey questionnaire about their experiences with recently introduced technologies. Participants were working in Dutch hospitals, psychiatric organizations, care organizations for mentally disabled people, home care organizations, nursing homes or homes for the elderly.

**Results:**

Half of the respondents were confronted with the introduction of a new technology in the last three years. Only half of these rated the introduction of the technology as positive.

The factors most frequently mentioned as impeding actual use were related to the (kind of) technology itself, such as malfunctioning, ease of use, relevance for patients, and risks to patients. Furthermore nursing staff stress the importance of an adequate innovation strategy.

**Conclusions:**

A prerequisite for the successful introduction of new technologies is to analyse determinants that may impede or enhance the introduction among potential users. For technological innovations special attention has to be paid to the (perceived) characteristics of the technology itself.

## Background

Many new technologies are becoming available within nursing care, such as home dialysis equipment or new infusion pumps that change the nursing staff's daily routines. In addition, all kinds of technologies that support distant care, such as telecare technology, have consequences for nursing practice. Another development is the introduction of electronic information systems such as electronic patient records. Technologies are aimed at increasing the quality of care, reducing healthcare costs or solving workforce problems [1]. It is widely recognised that one of the main problems with the introduction of innovations in general, such as technologies or clinical guidelines, is that professionals do not automatically use them as intended by the developers. This means that a substantial proportion of patients/clients will not receive the intended care in such a way that they benefit from these innovations. This article focuses on the determinants of a successful introduction of new technology in nursing care.

Several models and frameworks exist on how to introduce innovations in health care effectively [2-8]. By innovation, we mean, for example, guidelines, interventions or programs that are perceived as new by an individual or organisation [9]. Most models originate from the Diffusion of Innovations theory of Rogers. Despite some differences, all models follow a similar planning sequence: (1) the innovations should be introduced systematically to maximise success, and (2) a planned innovation strategy should be tailored to the determinants that facilitate or impede the intended innovation process.

For the current study we used a framework which was originally developed for analysing determinants of innovation processes in health care [3]. From 1999 to 2002 a literature review on determinants of innovation processes was performed in which only empirical studies (n = 57) were included. Subsequently, a Delphi study among 44 implementation experts (researchers, programme managers, and consultants/advisors) was conducted to achieve consensus on the determinants identified from the literature review. The results of the literature review matched those found in the Delphi study. This resulted in a list of 50 potentially relevant determinants. Since 2002, the framework and the list of 50 determinants have been used for research on the introduction of several innovations in health care, in the Netherlands as well as abroad [10-13].

**Figure 1:**
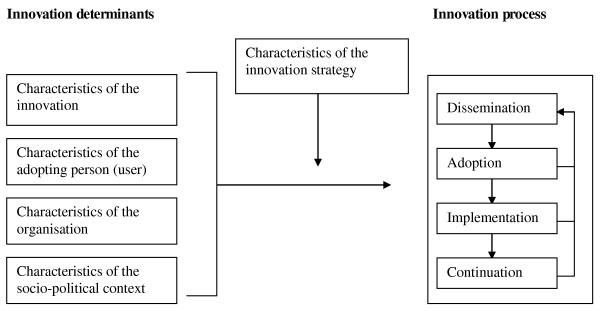
**Framework representing the innovation process and related categories of determinants derived from Fleuren et al**. [3]

The framework is presented in Figure 1. The right-hand section of Figure 1 shows the four main stages in innovation processes. Dissemination means that every professional is actually supplied with the innovation. At the adoption stage, the professional will develop a positive or negative intention to use the innovation. During the implementation stage, the professional tries to use the innovation in daily practice and experiences what working with the innovation means. Finally, there is the continuation stage, in which working with the innovation becomes routine practice.

The four main stages can be thought of as critical phases when the desired change may or may not occur. The transition from one stage to the next can be affected positively or negatively by various factors or "determinants" (left-hand section of Figure 1), which can be divided into four categories [2,3]:

1) the innovation itself - in this article the technology to be introduced; determinants are, for example, the involvement of potential users in developing the technology, the perceived complexity of the technology or the relative benefits it offers compared with the current situation. For example new technologies that are perceived as easy to use or compatible with the current working situation are more likely to be used.

2) the (potential) user of the technology - in this case nursing staff; user determinants include the knowledge or skills needed to use the technology, outcome expectations or perceived support by colleagues or management. For example nurses who feel capable (self-efficacy) of working with a new technology are more likely to use the innovation.

3) the organization; determinants include staff turnover, staff capacity, resource allocation or the decision making process in the organization. For example, sufficient time and staff availability will positively influence the uptake of new technologies.

4) the socio-political context; examples of determinants are rules, legislation, (anticipated) patient co-operation or (anticipated) patient satisfaction. For instance, structural funds that are made available at the early stage of the innovation process can boost the use of the new technology, or patients' willingness to cooperate.

Although the nursing staff play a crucial role in the innovation process, they do not work in isolation and are part of an organisation, which in turn is part of a larger environment. For these reasons, the characteristics of the organisation and the socio-political context in which the organisation operates should also be taken into account. This is also stressed by other authors [14-18].

In the model the perception of the (potential) user plays a crucial role. If e.g. a user says time-constrains is a problem for using the technology, so be it. Even if we could argue that this user has objectively the same amount of time compared to colleagues who do use the technology. If we ignore these perceptions we know that change will not occur. So, pointing out e.g. the technology is evidence-based or has no shortcoming will not alter the adoption or this specific user.

A detailed understanding of critical determinants is a prerequisite for designing an innovation strategy that can achieve real change (middle of Figure 1). If a determinant analysis is not conducted and/or the applied innovation strategy does not take the relevant determinants into account, the innovation process could fail [2-4,19]. One reason for failure is if the applied innovation strategy focuses on determinants that are irrelevant to the innovation process. Secondly, the chosen strategies may be inappropriate as a way of steering the relevant determinants of the innovation process. If for example nurses lack knowledge of how to use the technology, training might be a good innovation strategy. However, if time constraints are the issue, training will not solve this problem.

We applied these insights to the present study.

### Aim

The aim of this study is to gain a better understanding of the determinants influencing the success or failure of the innovation process of new technologies as perceived by nursing staff.

Research questions:

1. What kinds of technologies have recently been introduced in Dutch nursing care and how do nursing staff value the way these are introduced (innovation process)?

2. What determinants impede or enhance the introduction of new technologies according to nursing staff? More specifically, which are the determining characteristics of (a) the technology; (b) nursing staff members as (potential) users; (c) the organization, and (d) the socio-political context?

## Methods

### Participants

The study population is a nationally representative research sample of nursing staff in the Netherlands, further referred to as the Nursing Staff Panel. The Panel consists of a permanent group of Nursing Assistants (NAs) and Registered Nurses (RNs) who agree to fill in a postal questionnaire twice a year on average. In the Netherlands, NAs have three years of professional training. RNs are educated at two different levels and comprise nurses at associate degree level (3-3.5 years of professional training) and nurses at bachelor degree level (at least 4 years of professional training).

Candidates for the Nursing Staff Panel were recruited from a random sample of nursing staff registered with the National Employee Insurance Agency, where all Dutch employees are required to be insured. This Agency, which has the addresses of all employees in Dutch health care as well as their job titles, asked a random sample of nursing staff whether they were willing to participate in the Panel. The Panel coordinator (AdV) selected 1, 018 individuals to form the Nursing Staff Panel, making the selection in such a way that members represent the nursing staff in the largest health care sectors in the Netherlands, i.e. hospitals, psychiatry, care for mentally disabled people, home care, nursing homes and homes for the elderly. The age and gender of the selected group correspond to the age and gender of the population of Dutch nursing staff. This representative group makes up the Nursing Staff Panel. The questionnaire was sent to all 1, 018 members of the Panel in January 2009, followed by two reminders, about 14 and 28 days later.

A total of 685 panel members completed a postal questionnaire about the use of technology (response rate 67%). Most respondents (89%) delivered direct patient care exclusively, while 11% were also involved in management tasks (nursing staff members with only management tasks were excluded from the Panel). The respondents were employed to work 24 hours a week on average (standard deviation (*sd*) 8 hours) and had been working in care for an average of 21 years (*sd *9 years). Most respondents were female (90%). We compared the respondents and non-respondents and found no statistically significant differences in the percentages of females in both groups (p > .05), although we did find a statistically significant difference in age: the non-respondents were younger (average age 43 years) than the respondents (average age 45 years, *sd *9 years). Nonetheless, the Panel is largely representative of all Dutch NAs and RNs working in the Netherlands.

### Questionnaire

The questionnaire (additional file 1) addressed technologies that had been introduced in the past three years, such as sensors, electronic monitoring of medical data, telecare and electronic patient records. In order to obtain an overview of recently introduced new technologies, respondents were first asked whether a new technology had been introduced in the team during the previous three years (yes/no). If the answer was 'yes' the respondent was asked to write a short description of the new technology (open-ended question).

The evaluation of the introduction addressed three main issues. First, a forced-choice question was asked on how they evaluated the introduction process of the new technology (answered on a 5-point Likert scale, ranging from 'very good' to 'very bad'). Then, two open-ended questions were asked regarding what were the 1) enhancing factors in the uptake of the technology and 2) impeding factors in the uptake of the technology.

The face validity and content validity of the draft questionnaire were assessed individually by five experts in the field of nursing care: two researchers in this field, two individuals with considerable practical knowledge of working as a nurse, and one representative of the Ministry of Health. They were asked to comment individually on the face validity and content validity of the questionnaire and to judge whether the questions and possible answers were unequivocal. This resulted in minor modifications to the questionnaire. The questionnaire (in Dutch) can be obtained on request from the first author.

### Ethical considerations

As this was a questionnaire-based study with nursing staff and without patient involvement, no approval by an ethics committee is required in the Netherlands. Study participation was voluntary. Responses were anonymous and non-traceable to individual nurses.

### Coding and analyses

The new technologies that the respondents described encompassed a total of 25 different technologies, showing that nursing staff have experienced a diversity of new technologies. The authors discussed the differences and similarities of these technologies and decided to construct three main clusters. The first cluster comprises electronic information systems such as electronic patient records, digital nursing plans and electronic medical records. The technologies within this cluster involve digital data storage. The second cluster comprises technologies designed for distant care such as telecare and telemedicine. These technologies require the use of a computer in the communication between professional and patient. The third cluster concerns medical devices such as infusion pumps, heart defibrillators, heart rate monitors, and respiratory care devices. For the proper application of these devices a certain expertise is needed. The authors decided that the remaining technologies could not be further clustered. Examples are in/out touch systems that register the time the nurse is in the patient's room, washing without water (with disposable wash cloths), and a new call-for-help system.

The coding of the determinants was as follows. The answers to the two open-ended questions concerning determinants of the introduction process of 50 randomly chosen respondents were originally coded by two researchers (AdV and MF) independently. This resulted in an intercoder reliability of 81%. Differences in interpreting the codes were discussed until consensus was reached. Based on the discussions, the description of each code was made more precise and examples were added to the coding system. To maximize the reliability of the codes the researcher who coded the remaining answers (AdV) discussed the codes with another researcher (MF) whenever there was any doubt (approximately 10% of all codes). The coding system was based on the original list of 50 possible determinants of Fleuren et al. [3](see appendix). These determinants are clustered in the four main categories of determinants mentioned in the introduction (also Figure 1). However, while coding the data it appeared that the respondents often did not make a clear distinction between determinants related to the organisation or to the socio-political context. For instance, when they indicated there was a lack of money to implement the innovation it remained unclear whether the organisation spent too little money on the introduction or whether the organisation did not receive enough money in general from the government, insurance company or other stakeholders in order to introduce new technologies. Therefore these two categories were combined into a broad category 'organisational and political context'. This is in line with later publications of Fleuren et al. [20], in which the authors specify several determinants which are related to both the level of the organisation and the socio-political context. Respondents did not always focus only on determinants but also on the chosen innovation strategy. Of the 857 different elements mentioned by the respondents, 518 (i.e. 60.4%) are determinants and 339 (i.e. 39.6%) are characteristics of the innovation strategy. Elements related to the innovation strategies were separately coded using existing taxonomies where possible [10,21], and the results are presented in separate tables. The coding followed the same procedure as the determinants: after coding the answers on the two questions of 50 respondents the intercoder reliability was 86%. Subsequently one researcher (AdV) coded the answers and discussed doubtful codes with another researcher (MF).

For the answers to the questions descriptive analyses were used. Chi-square tests were performed to explore the relationship between the introduction and the determinants that influence the introduction and the kind of technology that was introduced as well as the health care sector in which the introduction took place. The analyses were performed using Stata 10.1.

## Results

### New technologies and evaluation of the introduction process

Nearly half of the respondents (45.4%, n = 311) had experienced the introduction of a technological innovation in the previous three years, each respondent describing one technology. Most frequently mentioned (37.3%) were electronic information systems. Introduction of distant care technology was cited by 14.2% of the respondents and 12.5% of the respondents referred to the introduction of a medical device. The other technologies (36.0%) were wide-ranging and could not be further classified into an umbrella category. There is a strong association between kind of technology and the health care sector of the respondent (chi square (12) = 116.76, p < .001). Respondents who mentioned electronic information systems most often (78.6%) worked in hospitals, psychiatry, nursing homes and homes for the elderly. This also applies to the not further classified examples (67.9%). The examples of distant care were mainly (70.4%) mentioned by those working in home health care, nursing homes and homes for the elderly. Most examples of medical devices (77.5%) were cited by respondents in hospitals.

When asked to give an evaluation of the introduction process for the new technology, half of the nursing staff perceived the introduction process as good or even very good (table 1). The evaluation is not related to the kind of technology introduced (chi square (12) = 14.27, p = .284, not in table), nor to the health care sector (chi square (16) = 16.67, p = .407, not in table)

**Table 1:** Nursing staff's evaluation of the introduction process of the technology (n = 307, 4 missing)

Evaluation	%
very good	4.6
good	46.6
moderate	32.2
bad	10.6
very bad	5.9
Total	100

### Impeding and enhancing determinants

A total of 518 determinants influencing the introduction process were mentioned. Twice as many were impeding (n = 347, 67.0%) compared to enhancing determinants (n = 171, 33.0%) (table 2).

**Table 2:** Number and percentage of determinants of the innovation process mentioned by nursing staff (n = 311) as enhancing and impeding in each category of determinants

Characteristics of the...	enhancing n	%	impeding n	%	total n	%
Technology	96	56.1	200	57.6	296	57.1
(Potential) user	30	17.5	67	19.3	97	18.7
Organisational and political context	45	26.3	80	23.1	125	24.1
Total	171	100	347	100	518	100

The table shows that most determinants relate to the technology itself (57.1% of all codes). Determinants relating to the organisation/context (24.1% of all codes) and the (potential) user, in this case nursing staff (18.7% of all codes), were mentioned more or less equally. Enhancing and impeding determinants were almost equally distributed among the three categories of determinants.

Chi square tests were used to explore relationships between the kind of new technology that was introduced (i.e. electronic information systems, distant care, medical devices, other technologies) and the perceived categories of relevant determinants of the innovation process.

Of the six chi square tests (for every category of enhancing and of impeding determinants), two resulted in a statistically significant test score (p < .05, not in table), implying a relationship between the kind of technology and the perceived enhancing determinants within the technology itself and impeding determinants within the organisational and political context. Respondents reporting about distant care devices more frequently (46.5%) referred to enhancing factors relating to the technology than respondents reporting about the other kinds of technologies (23.6%); specifically, they more often mentioned the relative advantages of distant care for the patients as a facilitating factor. Respondents who reported about the introduction of an electronic information system most often (35.1% versus 18.1% of the other respondents) mentioned impeding characteristics within the organisational and political context, such as not enough computers to use the new system.

No statistically significant relationships were found between health care sector and the categories of determinants mentioned by the respondents (no table).

Below, we will elaborate on the main categories of determinants.

#### Determinants related to the technology

Most remarks concerned the perceived (lack of) relative advantage of the new technology and technical shortcomings of the new technology (table 3). The uptake of technologies that were perceived as clearly advantageous for the nurse, was more straightforward. These advantages may be professional, financial, time saving, more job satisfaction etc. For example, nurses experienced considerable time saving with an electronic system for communicating lab results, rendering telephone calls unnecessary. On the other hand if nurses saw no advantage in the technology or even perceived disadvantages, they were generally less motivated to actually use it. One example is when nurses were given hand-held computers for sending and receiving information, linked to a central system. This was experienced as unpleasant since it substantially reduced personal contact with colleagues.

**Table 3:** Most frequently (at least 3% of the respondents or 10 times) mentioned enhancing and impeding determinants of the innovation process in each category of determinants (n = 311)

Domain	enhancing (n times mentioned, %)	impeding (n times mentioned, %)	total^1^ n	%
*technology*				
	- relative advantage: the technology is perceived as advantageous (48, 15.4%)	- relative advantage: the technology is not perceived as advantageous (51, 16.4%)	89	28.6
		- the technology is (still) dysfunctional, contains bugs (78, 25.1%)	78	25.1
	- the technology is easy to use (18, 5.8%)	- the technology is not easy to use (26, 8.4%)	44	14.1
	- the relevance of the technology for the patient is high (25, 8.0%)	- the relevance of the technology for the patient is low or not clear (13, 4.2%)	37	11.9
		- the technology carries risks for the patient compared with the existing situation (11, 3.5%)	11	3.5
				
*(potential) user*				
	- support from colleagues in using the technology (14, 4.5%)	- lack of support from colleagues in using the technology (15, 4.8%)	29	9.3
		- lack of skills needed to use the technology (28, 9.0%)	29	9.3
		- lack of support from other health professionals in using the technology (10, 3.2%)	13	4.2
				
*organisational and political context*				
	- nursing staff are involved in the development of the technology and/or innovation strategy (14, 4.5%)	- nursing staff are not (sufficiently) involved in the development of the technology and/or innovation strategy (24, 7.7%)	36	11.6
	- enough time available to adopt and use the technology (14, 4.5%)	- not enough time available to adopt and use the technology (12, 3.9%)	26	8.4
		- not enough resources made available to adopt and use the technology (e.g. equipment, manuals) (21, 6.8%)	23	7.4

^1^total number of respondents referring to the determinant as either impeding or facilitating. The total n is not always the sum of the numbers in the first and second column since (1) a determinant can be mentioned as both enhancing and impeding by the same person, (2) categories mentioned less than 10 times are not displayed in the first and second column

Dysfunctional technology was mentioned in 25.1% of the introduction processes as an impeding determinant. Clearly, nursing staff said not to use a new technology if it malfunctioned or if necessary functionalities were missing in their perception. Nursing staff often encounter such problems. An example mentioned was the introduction of new infusion pumps in a hospital for administering gyrostatic drugs. These pumps could not be used because an essential function was missing according to the respondent. A remote care system transpired to be unreliable since it did not always react when it was meant to. Nursing staff who visited patients at home referred to an electronic key system used to unlock the front door. In rainy weather this didn't always work and the nurse could not enter the patient's home.

If nurses perceived the technology as being easy to use, this enhanced the uptake of the technology. Conversely, if the technology was perceived as being difficult to use, this was cited as a barrier. For example, home telecare mainly consists of an audio-visual connection between the patient and professionals, generally nurses. Some nurses perceived the system as complex and difficult to learn. Therefore they were not very willing to use the technology.

The perceived (potential) relevance of the new technology for the patient was mentioned as playing a role in the success of 11.9% of the introduction processes, either as facilitating or as impeding its introduction. When nursing staff thought the patient would benefit from the new technology, they were more willing to actually use it. The opposite was also found: when the anticipated benefits for the patient were thought to be low or unclear, this impeded the introduction. Although the perceived negative consequences for the patients are sometimes only temporary and the benefits will prevail after some time, the introduction is nonetheless impeded according to the respondents. In the case of the above-mentioned electronic key systems, patients will clearly benefit because a nurse can enter the house if the patient is not able to open the door. However, initially the system had serious negative consequences for some patients because nurses were confronted with patients who were anxious that the system might not be burglar-proof.

Finally, if nursing staff believed the new technology carried risks for the patient (e.g. the patient's safety), the introduction process was sometimes impeded. This was the case in a nursing home ward where patient restraints such as belts, special sheets, and wrist straps were replaced by an electronic patient monitoring system combined with psychosocial interventions. Nursing staff who believed that patients were less safe in the new situation because of a higher risk of falling were less willing to change their work practices.

#### Determinants related to the (potential) user

The respondents named three determinants related to nursing staff as being either impeding or enhancing (table 3 second block). Most frequently mentioned (in 9.3% of the introduction processes) were the perceived support from colleagues and the skills needed to use the technology. If colleagues did not support the new technology, the use of the technology was more difficult. 'Enthusiastic colleagues' were reported as being a very stimulating for the adoption and use of new technology. If skills were perceived as being inadequate, the innovation process was more likely to be delayed. Some nursing staff reported having insufficient skills to handle technologies, e.g. they felt not able to manage using a computer mouse properly.

In addition to perceived support from colleagues, a perceived lack of support from other health professionals was reported as an impeding determinant.

#### Determinants related to the organisational and political context

Respondents cited the involvement of nursing staff in the decision making process as being influential to the introduction of new technology (table 3, third block). Authoritative decisions (e.g. making the use of the technology compulsory) were reported to reduce the likelihood of success. This also applies to technologies that are developed without - in the eyes of the respondents-consulting the nursing staff about their needs and wishes. The opposite was likewise true: if nursing staff felt or were included in the development or choice of new technology and in the design of the innovation strategy, the innovation process was perceived as more successful.

Another determinant was the perceived time available to adopt and use the new technology. If there is enough time, nursing staff can practice with the new technology. Not enough time available for training and practice was thought to impede the introduction.

Finally, a lack of resources was sometimes mentioned as a problem. In the case of an electronic patient record system there were only a few computers available to consult the system. As a result, information was not always available and patient records were not completed. A lack of hand-held computers was also cited.

### The innovation strategy

As already alluded to in the methods section the respondents (n = 311) cited 339 characteristics of the innovation strategy that influenced the introduction of the new technology. Most of the characteristics (n = 216, 63.7%) were described as facilitating. The remaining characteristics (n = 123, 36.3%) were described as impeding. Table 4 shows the characteristics mentioned.

**Table 4:** Most frequently (at least 3% of 311 respondents or 10 times) mentioned enhancing and impeding characteristics of the innovation strategy

enhancing (n times mentioned, %)	impeding (n times mentioned, %)	**total**^**1 **^**n**	%
- training and coaching (131, 42.1%)	- no or inadequate training and coaching (52, 16.7%)	175	56.3
- support system, help-desk (31, 10.0%)	- no support system, helpdesk (13, 4.2%)	42	13.5
- opportunities to evaluate the introduction, possibility to share experiences (18, 5.8%)	- no/few opportunities to evaluate the introduction, possibility to share experiences (15, 4.8%)	33	10.6
	- no adequate time frame, planning of the process (27, 8.7%)	31	10.0
- availability of simple, effective instruction materials (13, 4.2%)		17	5.5
- active promotion of the new technology (10, 3.2%)		13	4.2

^1^the total n is not always the sum of the numbers in the first and second column since (1) a category can be mentioned as both enhancing and impeding by the same person, (2) categories mentioned less than 10 times are not displayed in the first and second column

Training and coaching is the most frequently mentioned factor associated with the successful introduction of a technological innovation (referred to by 56.3% of the respondents). If the training and coaching were perceived to be adequate this is relatively frequently cited as a facilitating factor. It was found important for example that attention is paid to how the technology can be used in daily work routines. When pro-active visits were made to nursing staff to answer possible questions about the new technology, this was highly valued. 'Train the trainer' concepts are mentioned as dangerous since insufficient application and faults are easily spread.

If a technology was accompanied by a help-desk or other kind of support system, the innovation was perceived to be used more easily. If the technology falters, it is important to have someone who can quickly solve the problem.

Nursing staff also referred to the importance of opportunities to evaluate the introduction, to share their experiences and to receive adequate feedback about the use and consequences of the new technology. If nursing staff are experiencing problems and this goes unnoticed by the organisation there is a high risk of resistance to the new technology.

What went wrong in the opinion of some respondents was the time frame of the introduction of the technology. For example the planning of training activities in relation to the use of the new technology. Sometimes several weeks or even months elapsed between training and the actual time when the technology was available for use in daily practice. The opposite was also mentioned: the technology was already available but nursing staff were not yet trained to use it properly. Some respondents mentioned that there was no time frame at all.

Easily accessible materials are valued by nursing staff. For example a small user-guide showing the step-by-step procedure to be followed for a particular action.

Finally, respondents mentioned that the presence of leading figures who actively promote the new technology facilitates the introduction and is highly valued by nursing staff.

## Discussion

### Strengths and limitations of the study

In the framework we used the perception of the (potential) user of a technology plays a crucial role. When e.g. dysfunctional technology is mentioned as a barrier for use, this could either mean the technology has real shortcomings (objectively) or in the eyes of the user (subjectively). In our view the perception should always be the starting point for change. Because even if we could argue that the technology technically functions well, this would not alter the uptake of the technology in most cases. This also implies that when introducing new technologies more detailed information is needed (see below). The present study only offers insight in how a large representative group of nurses perceives new technology.

Second, the determinants were derived from experiences with a broad variety of technologies in a wide range of health care settings. As a consequence, the determinants found in this study may be assumed to be generally valid. The results of the explorative analyses and also the framework/model we used suggest that in more specific settings and with a particular technology some determinants might play a more prominent role than others. In each new situation, therefore, one should analyse the determinants that could play a role in the innovation process in order to design an adequate innovation strategy for that specific context and technology and the intended users.

Third, as the study explores the perceptions of nurse-users this implies a strong emphasis on impeding and facilitating determinants on a user level. Managers, for example, may more frequently refer to determinants on a more distant, organizational and political level if they are asked the same question. However, the strength of this study is that it examines determinants as perceived by the (intended) users of new technologies. Because all respondents had experienced the introduction of a new technology, the determinants cited can be considered to be valid.

Fourth, in our questionnaire, we did not differentiate between the stages of dissemination, adoption, implementation and continuation. Fleuren et al. [3] indicated in their study at which stages which determinants might be particularly relevant. Since the questionnaire addressed innovations that were introduced in the past three years, we felt respondents would not be able to recall reliably which determinants were particularly valid in the several stages. Besides, in daily practice the stages of adoption and implementation as well as the stages of implementation and continuation sometimes overlap. However, when coding the answers, we felt that most determinants were related to the stage of implementation.

### Perceived dysfunctional technologies

Most identified determinants are related to the characteristics of the technology. Perceived dysfunctional technologies and bugs are the most frequently cited impeding determinants related to the technology itself. Difficulties with (using) the technology itself is also found in other studies on nurses' experiences with new technologies [22,23]. The results differ from studies on determinants of social innovations such as medical guidelines or health promotion programmes, where most determinants are related to characteristics of the user [2,3].

One possible explanation might be that there is a logical sequence in the importance of determinants. Characteristics of the user, such as skills or knowledge, may be perceived as less relevant when the technology itself - objectively or subjectively- fails. Therefore, technologies should be properly tested by developers and piloted in the organization by the potential users before they are widely introduced to nurse-users. Starting off with - perceived- dysfunctional technology can also severely affect the motivation of nursing staff with regard to getting to know the new technology, and this initial bad impression can eventually obstruct the adoption decision. Nursing staff are especially concerned about the safety of their patients [1,24]. Dysfunctional technologies may harm the patient. Therefore, innovations that are still dysfunctional after testing should not be implemented; in such cases the technology has first to be improved and optimized.

### Determinants of successful implementation

Besides the above mentioned malfunctioning, the current study also stresses the importance of the perceived relevance of the new technology for the patient, as well as the relative advantage in the uptake of the technology by the nursing staff.

More than half (56%) of the nurses indicate that they would like more involvement by nurses in the development of the technology and/or the innovation strategy [1]. Remarkably, the involvement of nursing staff in the innovation strategy was not often spontaneously mentioned as a determinant of successful implementation. This could imply that the involvement of nurses in innovation processes has been low and has therefore led to a mismatch between determinants and innovation strategy and consequently, to a rather negative evaluation of the introduction of new technologies by the nurse-users. Implementation theories and research indicate that involving future users early on in the innovation process is a condition *sine qua non *[2,9,14,23].

Finally, respondents often stressed the importance of training and coaching, which corresponds to the impeding role of lack of skills. However, training and coaching are usually not sufficient when implementing innovations; multifaceted strategies often appear to be more effective [17,25]. Besides, training and coaching will not solve the problem of dysfunctional technology. So, first the technology should be properly tested and piloted in the organization. In addition, training, coaching and the presence of a help-desk or support system facilitate the adoption and use of a new technology [26]. After the initial introduction nursing staff want opportunities to share their experiences and evaluate the introduction. A strategy based on an analysis of determinants with a well-considered time schedule will positively influence the innovation process.

These determinants are also found in models that are tailored to the individual acceptance and usage of information systems such as the widely used technology acceptance model (TAM) [27,28] and the more recently introduced Unified Theory of Acceptance and Use of Technology (UTAUT) [29]. For instance, in TAM the core determinants are perceived usefulness and perceived ease of use. Relative advantage and relevance for the patient correspond to perceived usefulness in TAM, and ease of use is also a concept in TAM. Other empirical findings also show that the individual's perceptions of the characteristics of a technology are important determinants of technology acceptance [30,31].

In the case of mandatory tasks, which usually apply to nurses, the TAM includes the subjective norm as a predictor for intention to use a technology [29]: this corresponds to the role of colleagues and other health professionals in the innovation process in our study. Perceived support from colleagues or other health professionals was cited frequently. Obviously, nursing staff need collegial support when using a new technology. In cases where the new technology is to be used in multidisciplinary health care settings, such as hospitals, support from other professional disciplines (e.g. doctors) is also necessary. Although there is little evidence of the importance of team characteristics and team directed strategies in relation to the introduction of innovations, particularly in nursing [32], research suggests that these may be relevant in a general sense [25].

### Recommendations

On the basis of the conclusions and critical reflection, we would like to make some recommendations. First, nursing staff should be more involved in the first place in order to understand whether the technology has any relevance for the user or the end-user (patient/client), and subsequently when it is developed. This is a condition *sine qua non*, which is generally known but often omitted due to lack of financial recourses or time constraints.

Second, as nursing staff frequently mentioned malfunctioning technologies, this means that new technologies should be thoroughly pilot-tested in daily practice before they are even introduced. Technologies should be pilot-implemented in daily practice. This is the standard procedure for many clinical guidelines. A determinant analysis is performed with the final draft of the guideline by asking the potential users for example to "test" the final draft in daily practice for a brief period of time. The guidelines are adapted to the results and the results show what innovation strategies should be developed for national dissemination, adoption and implementation.

Third, involving nursing staff in analysing which specific determinants play a role is important to enhance the likelihood of addressing the right determinants. As these determinants are identified by the nurse-users themselves, they may be able to have a profound influence on the success of the innovation process. Many theories can provide a starting point for developing innovation strategies to change the determinants that have been shown to be relevant for the successful introduction of the technology [4], and these should be prioritised. Subsequently, the potential users, in this case nursing staff, should be involved in organising and operationalising the theory into practical strategies. Involving nursing staff, for example in choosing the kind of training required is crucial, since nurses are best at indicating what they don't know or which important skills they are missing.

## Conclusion

The article offers insight in how a large group of nurse-users perceives new technologies. The primary conclusion is that the introduction of new technologies is common practice in nursing care in the Netherlands, as half of our respondents experienced the introduction of a technological innovation in the last three years. Electronic information systems, technologies for providing distant care and medical devices were the new technologies most often mentioned by nursing staff.

A second conclusion is that there is still a long way to go in regard to managing the introduction of new technologies, as only half of the introduction processes were positively rated by nursing staff.

Thirdly, it can be concluded that characteristics of the technology itself were the most frequently cited determinants impeding actual use. The characteristics of the nurses who use the new technology and the organisational and political environment are also perceived to play a role in either facilitating or impeding the uptake of new technologies.

## Competing interests

The authors declare that they have no competing interests.

## Authors' contributions

AdV and AF were the principal investigators and were responsible for the study concept and design, interpretation of data and preparation of the manuscript. NB was responsible for the analyses. MF and AdV coded the answers on the open ended questions. MF and NB wrote parts of the manuscript. All authors read and approved the final manuscript.

## Appendix: Description of the determinants*

Source: Fleuren et al. [3]

### Determinants related to the innovation**

1 Extent to which the procedures/guidelines of the innovation are clear

2 Compatibility: degree to which the innovation is perceived as consistent with existing work procedures

3 Trialabitity: extent to which the innovation can be subjected to trial

4 Relative advantage: extent to which the innovation is perceived as advantageous

5 Observability: degree to which the results of the innovations are observable to the health professional

6 Extent to which the innovation is appealing to use

7 Relevance of the innovation for the patient: extent to which the innovation has added value

8 Extent to which the innovation carries risks to the patient compared with the existing situation

9 Frequency of using the innovation: high, low

10 Health professionals are involved in the development of the innovation

### Determinants related to the adopting person/user/health professional

11 Support from/of colleagues in implementing the innovation

12 Support from/of other health professionals in implementing the innovation

13 Support from/of their supervisors in the department/organisation as to the implementation of the innovation

14 Support from/of higher management in the organisation as to the implementation of the innovation

15 Extent to which colleagues implement the innovation (modelling)

16 Extent to which the health professional has the skills needed to implement the innovation

17 Extent to which the health professional has the knowledge needed to implement the innovation

18 Self-efficacy: confidence to perform the behaviour needed to implement the innovation

19 Extent to which ownership by the health professionals is perceived

20 Extent to which the innovation fits the perceived task orientation of the health professional

21 Extent to which the health professional expects that the patient will co-operate in the innovation

22 Extent to which the health professional expects that the patient will be satisfied with the innovation

23 Extent to which the health professional suffers from work-related stress

24 Extent to which goals of health professionals with respect to the innovation are contradictory

25 Extent to which the health professional has ethical problems with the innovation

### Determinants related to the organisation

26 Decision making process and procedures in the organisation: top-down or bottom-up/participatory

27 Hierarchical structure: extent to which decision making process is formalised through hierarchical procedures

28 Formal reinforcement by management to integrate innovation into organisational policies

29 Organisational size (number of employees): large, medium size, small

30 Functional structure (task oriented) versus product structure (output oriented)

31 Relationship with other departments or organisations: inward-looking or outreaching

32 Nature of the collaboration between departments, being involved in the innovation

33 Staff turnover: high, average, low

34 Degree of staff capacity in organisation or department which implements the innovation

35 Available expertise, in relation to the innovation in the organisation or department

36 Logistical procedures related to the innovation, e.g. logistical problems in scheduling patients

37 Number of potential users to be reached: many, few

38 Financial resources made available for implementing the innovation **

39 Reimbursement for health professionals/organisations to facilitate extra effort in applying the innovation **

40 Other resources made available for implementing the innovation (e.g. equipment, manuals) **

41 Administrative support available to the users (health professionals) of the implementation

42 Time available to implement the innovation

43 Availability of staff responsible for co-ordinating implementation in the organisation/department

44 Opinion leader who influences opinions of others in the organisation or department (not the co-ordinator)

### Determinants related to the socio-political context

45 Willingness of the patient to co-operate with the innovation

46 Degree to which the patient is aware of the health benefits of the innovation

47 Patient doubts concerning the health professional's expertise and competence with respect to the innovation

48 Financial burden of the innovation imposed on the patient (e.g. no insurance cover)

49 Patient discomfort (physical or emotional) as a result of the innovation

50 The extent, to which the innovation fits into existing rules, regulations and legislation

* For the purpose of the present study extended by a new determinant 'Functionality of the technology (malfunctioning, bugs)'

** These determinants can also be classified as determinants related to the socio-political context

## Pre-publication history

The pre-publication history for this paper can be accessed here:

http://www.biomedcentral.com/1472-6947/11/67/prepub

## Supplementary Material

Additional file 1 **Part of the questionnaire on the introduction of new Technologies. Nursing Staff Panel**. Translation of the questions answered by the respondents. Click here for file
