# Supplementary material for: Successful implementation of new technologies in nursing care: a questionnaire survey of nurse-users
Source: BMC Med Inform Decis Mak. 2011 Oct 27;11:67. doi: 10.1186/1472-6947-11-67 (PMC3214145; doi:10.1186/1472-6947-11-67)
Supplement: Additional file 1 — Part of the questionnaire on the introduction of new Technologies. Nursing Staff Panel. Translation of the questions answered by the respondents. [file 1472-6947-11-67-S1.DOC]

Part of the questionnaire on the introduction of new Technologies. Nursing Staff Panel

January 2009, pages 22-23

A.J.E. de Veer & A.L. Francke, NIVEL, Utrecht, The Netherlands (a.deveer@nivel.nl)

| Your experiences with the introduction of new techniques and technologies |
| --- |

1. If a new technique or technology has been introduced in the last three years, we are curious to know how the introduction is evaluated. What went well and what went less well. Can you mention a new technique or technology in your team that in the last three years has been launched?
   - yes, namely (please give a short description of the technique or technology)…………….

……………………………………………………………………………………………..

……………………………………………………………………………………………..

……………………………………………………………………………………………..

……………………………………………………………………………………………..

……………………………………………………………………………………………..

……………………………………………………………………………………………..

……………………………………………………………………………………………..

……………………………………………………………………………………………..

- - no 🡪 go to question 60

1. What did they want to achieve with this new technique or technology? You can tick more than one reason.
   - better quality of care for patients
   - better quality of life of patients
   - empowering patients
   - safer care for patients
   - lower costs
   - labour saving, same nursing staff is able to care for more patients
   - reducing the physical demands of nursing staff
   - reducing the working pressure of nursing staff
   - increasing the attractiveness of working as a nurse
   - reducing the administrative tasks of nursing staff
   - other, namely …………………………………………………………………………….
2. How would you evaluate the way this new technique or technology was introduced?
   - very good
   - good
   - moderate
   - bad
   - very bad
3. Will you describe what went *well* and explain why?

…………………………………………………………………………………………………….

……………………………………………………………………………………………………..

……………………………………………………………………………………………………..

…………………………………………………………………………………………………….

……………………………………………………………………………………………………..

…………………………………………………………………………………………………….

……………………………………………………………………………………………………..

1. Will you describe what went *less well* and explain why?

…………………………………………………………………………………………………….

……………………………………………………………………………………………………..

……………………………………………………………………………………………………..

…………………………………………………………………………………………………….

……………………………………………………………………………………………………..

…………………………………………………………………………………………………….

…………………………………………………………………………………………………
